# Extracellular Matrix Regulation of Stem Cell Behavior

**DOI:** 10.1007/s40778-016-0056-2

**Published:** 2016-07-07

**Authors:** Maqsood Ahmed, Charles ffrench-Constant

**Affiliations:** MRC Centre of Regenerative Medicine, University of Edinburgh, 5 Little France Drive, Edinburgh, EH16 4UU UK

**Keywords:** Extracellular matrix, Stem cells, Stem cell niche, Microenvironment, Regeneration

## Abstract

Stem cells hold great promise in treating many diseases either through promoting endogenous cell repair or through direct cell transplants. In order to maximize their potential, understanding the fundamental signals and mechanisms that regulate their behavior is essential. The extracellular matrix (ECM) is one such component involved in mediating stem cell fate. Recent studies have made significant progress in understanding stem cell-ECM interactions. Technological developments have provided greater clarity in how cells may sense and respond to the ECM, in particular the physical properties of the matrix. This review summarizes recent developments, providing illustrative examples of the different modes with which the ECM controls both embryonic and adult stem cell behavior.

## Introduction

Stem cells are specialized, undifferentiated cells capable of self-renewing or differentiating into more tissue-specific cells. The earliest stem cell can be found in the embryo shortly after fertilization of the egg and has the ability to produce every other cell of the body, with the in vitro product of these cells being embryonic stem (ES) cells. As development continues, these pluripotent stem cells disappear giving rise to more lineage-restricted, tissue-specific, somatic stem cells which reside in specialized microenvironments termed “niches.” The niche refers to a specific anatomical location which is able to maintain cell stemness throughout life [1]. The resident somatic or adult stem cells are responsible for replacing cells that die either through natural wear and tear (homeostasis) or as a response to injury. The decision to lie dormant, self-renew, or differentiate is a consequence of the diverse cocktail of signals provided by the stem cell niche. How these niches develop and establish themselves is still an area of active research, particularly as there is great diversity in niche design. For example, muscle stem cells are distributed throughout the skeletal system as quiescent individual cells attached to the basal lamina of muscle fibers. Meanwhile, in many other cases, small substructures are established within tissues which house clusters of stem cells such as the subventricular and subgranular zones of the brain and in the bulge of the hair follicle. Despite these obvious differences, there are some common features of many stem cell niches. They contain heterogeneous cell populations consisting of both tissue-specific and generic cells such as endothelial cells, participating in often complex, bidirectional signaling; the immune system delivers dynamic regulation particularly during inflammation and tissue damage; and the presence of extracellular matrix (ECM) proteins—collagens, glycoproteins, proteoglycans, and glycosaminoglycans (Table 1)—which self-assemble in the interstitial spaces between cells or as basement membranes [39].

**Table 1:** Examples of extracellular matrix proteins not discussed in the main text, with a brief structural description and their known functions in regulating stem cells

ECM protein	Description	Function	Reference
Abi3bp	Contains Fn type III domain. Binds to heparin and collagen, assembles in the extracellular space	Promotes cell attachment. LoF inhibits MSC and CPC differentiation and increases proliferation	[2, 3]
Agrin	Major PG of the BM with 3 potential heparin sulfate attachment sites	Controls survival and proliferation of HSCs, LoF impairs hematopoiesis.	[4]
Cochlin	Present in the basilar membrane mirroring distribution of Fn, extensive homology to collagen binding domains of vWF	Promotes ESC self-renewal, inhibits neural differentiation, downstream BMP target	[5, 6]
CCN	6 members of CCN protein family characterized by having 4 cysteine rich domains	CCN1-3 principally involved in osteogenesis, chondrogenesis, and angiogenesis	[7–9]
Decorin	Small leucine-rich proteoglycan, interacts with collagn fibrils.	Interacts with a number of signaling pathways. Been shown to regulate kidney, muscle, hematopoietic, and neural stem cells.	[10–15]
IGFBP	Carrier proteins for insulin-like growth factor, six isoforms with 50 % homology	Crucial role in cardiac differentiation, regulator of hematopoietic and mesenchymal stem cells	[16–20]
Netrins	Structurally resemble laminins, 3 secreted proteins and 2 are membrane bound	Stem cell migration and involved reprogramming and maintaining pluripotency	[21]
Osteopontin Perlecan	Heparin sulfate proteoglycan present in basement membranes	Required for proliferation of intestinal stem cells and controls response of neural stem cells to growth factors	[22, 23]
Reelin	Secreted glycoprotein expressed in the brain	Required for multiple neural stem cell functions: migration, differentiation, and proliferation	[24–28]
Slits	Three isoforms with four leucine-rich repeat domains.	Promote neural stem cell proliferation and senescence of mammary stem cells	[29–32]
R-Spondins	Secreted proteins, with furin-like repeats and a thrombospondin domain	Potent WNT agonists involved in maintaining intestinal stem cells	[33, 34]
Tenascins	Glycoproteins with 14 EGF-like repeats and 8 or more Fn-III domains. Four isoforms.	Multiple functions in neural, hematopoietic and skin niches.	[35–38]

*BM* basement membrane, *CPC* cardiac progenitor cells, *HSC* hematopoietic stem cell, *IGFBP* insulin-like growth factor-binding protein, *LoF* loss of function, *MSC* mesenchymal stem cells, *PNS* peripheral nervous system, *PG* proteoglycan, *vWF* von Willebrand factor

The ECM is a multifunctional network of fibrous, gel-like material distributed throughout the body providing structural and biochemical support to all tissues. Matrix proteins have been implicated in many cellular processes ranging from dynamic behavior such as migration and morphogenesis as well as cell-fate decisions including proliferation, differentiation, and apoptosis [40]. The mechanisms with which ECM proteins can influence such a variety of functions are mediated by a large diversity of cell receptors, such as integrins, which can bind to some of the matrix components. In addition to providing instructive signals through direct ligand interactions, the ECM can signal in more indirect methods by sequestering growth factors and morphogens, acting as a local reservoir that can be released in response to changes in physiological conditions. ECM engagement can also potentiate other signaling pathways thereby providing a means with which cells can coordinate the barrage of incoming signals in the local milieu. More recently, stem cells have been shown to be able to sense and respond to the mechanical properties of the matrix. A further feature of the ECM is its highly dynamic nature. Cells can respond to microenvironmental cues and change ECM expression resulting in a rapid remodeling of the matrix in both the nature and quantity of constituent molecules. Alternatively, the cell can change the repertoire of matrix receptors it is expressing thereby providing a means for the intrinsic regulation of their interaction with the local microenvironment. In this way, a bi-directional signaling center is created which is able to evolve and exert differing effects over time and in response to multiple cues.

**Fig. 1:**
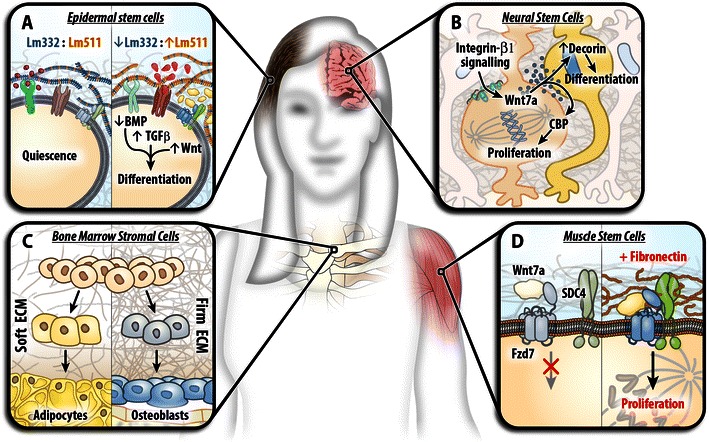
An overview of the extracellular matrix functions—direct, indirect, and biophysical—in stem cell niches distributed throughout the body. **a** In the adult epidermal stem cell niche, the basement membrane consists of laminin 332 and 511. When the precise ratio of laminin isoform is disrupted, BMP signaling is suppressed whilst TGFβ and Wnt signaling is amplified resulting in differentiation of the stem cells and niche depletion demonstrating the importance of matrix stoichiometry. **b** In the developing brain, β1-integrin signaling promotes Wnt7a secretion which acts non-cell autonomously to promote neurogenesis via decorin. **c** Bone marrow progenitor cells exposed to different biophysical environments differentiate in a stiffness-dependent manner with adipocytes generated on soft substrates and osteoblasts on stiff substrates. **d** After injury, muscle stem cells secrete fibronectin which acts autologously to promote stem cell expansion by binding to its receptor syndecan-4 and forming a complex with the Wnt receptor, Fzd7, and its ligand Wnt7a. In this way, fibronectin potentiates Wnt signaling

A number of transcriptional studies have identified ECM genes as critical, stage-specific, regulators of stem cell function [41–45]. This review provides a brief overview of the diverse ECM functions and highlights how ECM proteins contribute to stem cell regulation in both the embryonic and adult niche. Both direct and indirect signaling mechanisms are discussed followed by a brief overview of the biophysical effects of the ECM on stem cell function (summarized in Fig. 1).

## Direct ECM-stem Cell Interactions

The importance of ECM in the embryonic niche was demonstrated in early loss-of-function studies where the deletion of key ECM proteins such as fibronectin (Fn), collagen, and laminin (Lm) resulted in embryonic lethality [46]. Lms are a high-molecular weight, trimeric family of glycoproteins, and are the earliest expressed matrix proteins, detected at the two-cell stage of the developing embryo. They consist of an α-, β-, and γ- chain found in five, three, and three genetic variants, respectively, resulting in 16 different combinations in humans creating considerable tissue heterogeneity. Lms are named according to their chain composition; for example, Lm221 consists of α2, β2, and γ1 chains. Expression patterns of the individual Lm isoforms are controlled in a spatio-temporal manner as well as in tissue-specific locations. The inner cell mass of the human embryo consists of Lm511 and 521 that are able to engage the integrin receptor, α6β1, expressed on ES cells [47]. Recombinant Lm511/521 proteins have been shown to support the clonal survival and self-renewal of ES cells in vitro providing chemically defined, xeno-free conditions for the expansion and culture of ES cells suitable for clinical translation [48, 49]. Direct binding and activation of integrin α6β1 by Lm511/521 was able to trigger the PI3K/Akt pathway, which has been shown to block anoikis. Lm521 was found to be the superior of the two isoforms for ES renewal; however, the biological role of the Lm β-chain was not identified.

Many of the adult stem cell niches are also rich in Lms. The basement membrane of the epidermis consists of Lm332 and 511 with epidermal stem cells shown to express the integrins, α2β1, α3β1, and α6β4 [50, 51]. A recent study demonstrated that the precise ratio of Lm332/Lm511 in the hair follicle was critical for maintaining stem cell homeostasis [52]. Integrin-linked kinase (ILK), an actin-binding regulatory protein, was found to be a key regulator of the ECM microenvironment, responsible for the deposition of Lm332 and Lm511 gradients [53]. Using an ILK-deficient mouse model, the authors reported an abnormal Lm332/Lm511 ratio that led to a loss of quiescent stem cells through enhanced differentiation. Low TGFβ and Wnt activity and high levels of BMP characterize a quiescent state [54]. Lm511 was found to promote TGFβ2 signaling whilst Lm332 suppressed Wnt-β-catenin activity. ILK-deficient mice displayed reduced levels of Lm332 and an increase in Lm511, which led to an activated state with TGFβ2 and Wnt-β-catenin upregulated and BMP signaling suppressed resulting in aberrant hair follicle stem cell (HFSC) activation [52]. The study demonstrates the importance of stoichiometry between matrix molecules in regulating the niche steady state and homeostasis. It is also an example of reciprocal signaling between the matrix and stem cells where the stem cells are able to modulate niche composition, which can then affect function. A further example of this is the deposition of nephronectin by a subset of HFSCs creating a specialized microenvironment for α8 integrin-positive mesenchymal cells, which are then able to differentiate into smooth muscle cells [55]. Alternatively, collagen XVII (COL17a1) deposition by HFSCs provides a niche for the self-renewal and maintenance of not only HFSC but also melanocyte stem cells [56]. In this way, through the differential expression of ECM proteins by the HFSCs, considerable tissue heterogeneity is achieved. The presence of COL17a1 in the HFSC niche was also found to be critical in coordinating the hair follicle aging process [57••]. DNA damage accrued during aging results in proteolysis of COL17a1. Reduced COL17a1 levels in the niche leads to a loss of stemness of the HFSC and epidermal lineage commitment depleting the stem cell pool and shrinking the hair follicle. The aging process can be recaptured in COL17a1-deficient mice and prevented through the forced maintenance of COL17a1 suggesting stem cell aging is central to the tissue aging program and is mediated by the niche ECM.

The subventricular zone (SVZ) of the lateral ventricle is one of two stem cell niches in the adult brain, containing a population of neural stem cells (NSCs) that are able to self-renew and differentiate throughout life. NSCs and their progeny are anchored to the ECM emanating from blood vessels and the parenchyma of the ventricular cavity [58]. The composition of the ECM was found to be rich in Lms compared with the surrounding non-neurogenic areas [59]. On examining the expression of ECM receptors on the cells populating the niche, quiescent NSCs were found to be β1 integrin-negative whereas the dividing progenitors were β1 integrin-positive. When the niche was depleted of its progenitors, the NSCs became active in an effort to repopulate the niche, which was associated with an upregulation of β1 integrin expression [59]. This study suggests that the NSCs and progenitors are regulated not only by the extrinsic signals from the microenvironment but also by intrinsic mechanisms that coordinate their interactions with their surroundings.

## Indirect ECM-stem Cell Interactions

Soluble ligands such as fibroblast growth factor (FGF), bone morphogenetic protein (BMP), transforming growth factor beta (TGFβ), sonic hedgehog (SHH), and Wnt are potent signaling molecules able to induce specific cellular responses depending on their local concentration. The precise mechanism with which these diffuse morphogens are able to elicit their effects at the correct time and place needed to establish complex developmental patterns are still to be established. ECM molecules such as heparan sulfate proteoglycans (HSPGs) have been implicated in facilitating morphogen signaling by controlling the stability, movement, and presentation of growth factors [60]. HSPGs are composed of sulfated glycosaminoglycans attached to a core protein. Through a multistep process in the Golgi apparatus, heparan sulfate (HS) side chains are synthesized on the core protein. The HS chains are then elongated through the addition of 25–100 repeating disaccharide units —a process catalyzed by Ext1 and Ext2 proteins—followed by *N*-deacetylase/*N*-sulfotransferase (NDST)-mediated sulfation of the HS side chains [61]. The length of the HS side chains as well as the positioning and extent of sulfation contributes to the great diversity and specificity of HSPG-morphogen interactions [62].

The early stages of specification of the blastocyst and pluripotent stem cells into the three germ layers are driven in part by the FGF/Erk signaling pathway [63]. Mutations in genes responsible for the synthesis of HS provide genetic evidence for the necessity of HSPG for FGF signaling and normal mouse development [64]. Ext2 null mice, in which HS side chains are not synthesized, do not respond to FGF signaling and consequently fail to develop properly, lacking an extraembryonic ectoderm and mesoderm [65]. The HS chains were found to be crucial for local retention of FGF4 and FGF8 ligands. Meanwhile, undersulfation of mouse ES cells restricted their differentiation potential in vitro. An NDST1 and NDST2 double knock-out results in early embryonic lethality; however, ES cells from the blastocyst have been used to assess developmental potential [66]. Whilst the NDST1^−/−^NDST2^−/−^ mES cells are able to take initial steps towards all three germ layers, they were only able to differentiate into mesodermal tissue, albeit with a lower efficiency than wild type [67]. Neural differentiation was completely blocked with the cells stalling in a primitive ectoderm state, a phenotype that could be rescued with the addition of exogenous heparin suggesting HSPGs are required for neural induction.

Following neural induction, the rapid expansion of the neural progenitor pool is a hallmark of mammalian brain development [43]. In comparing the transcriptomes of rapidly dividing neural precursors in the human and mouse central nervous system (CNS), ECM genes, including distinct sets of collagens, laminins, and proteoglycans, were heavily expressed [43, 68]. Proteoglycans can determine the functional response of morphogen binding. SHH has been reported to have dual roles in embryonic and postnatal development, responsible for both proliferation and tissue patterning [69]. In an elegantly designed study, an N-terminal motif of SHH that mediates SHH-HSPG interactions was mutated without disrupting SHH’s affinity for its receptor, Patched [70, 71]. This allowed the investigation of SHH-HSPG interactions without interfering with other potential HSPG or SHH functions. The authors report that HSPG regulated the length of SHH signal and was required for neural stem cell proliferation but not for tissue patterning. Distinct gene expression profiles were obtained from the HSPG-dependent SHH signaling, suggesting proteoglycan binding does not just affect the presentation and concentration of morphogen but can regulate intracellular morphogen functions as well.

In the developing mouse brain, the α2 and α4 Lm chains appear to dominate in the ventricular zone and the overlying cortex [72]. Their principle receptor, β1 integrin, is also highly expressed, with the Lmα2-β1 integrin interaction being critical for maintaining the physical integrity of the embryonic cortical niche [73]. Loss-of-function studies to evaluate the precise role of these laminin-integrin interactions in the embryonic brain prove to be challenging due to architectural disruption and loss of adhesion. Gain-of-function studies, however, using a constitutively active β1 integrin has provided some mechanistic insight. Expression of β1 integrin resulted in an expansion of the neuroepithelia, but rather intriguingly, it is the neighboring, non-integrin expressing cells that were found to be the cause of this expansion suggesting a non-cell autonomous effect of β1 integrin signaling [10]. Transcriptional analysis revealed that the β1 integrin-expressing cells secrete Wnt7a that induces the expression of another ECM molecule, decorin, in the neighboring β1 integrin-negative cells. Decorin, a promiscuous small leucine-rich proteoglycan, has been reported to bind to a number of signaling molecules affecting a range of functions [74–76]. In this case, the effects of decorin were found to be through amplifying the TGFβ signaling pathway. Similar ECM mediated, non-cell-autonomous, mechanisms have been reported for specifying the mesendodermal and endodermal-ectodermal cell fates illustrating the crucial role ECM has in the spatial localization of signaling in cell specification during development [77, 78].

Similar indirect signaling mechanisms are at play in adult niches. For example, quiescent muscle stem cells (MuSCs, satellite cells) are activated in response to injury [79]. Using intravital two-photon imaging combined with second-harmonic generation microscopy, time-lapse images of the muscle regeneration process in live mice was directly visualized [80•]. ECM remnants from injured skeletal muscle fibers were found to act as templates for regeneration with the intravital imaging providing direct visual evidence for the role of ECM in promoting regeneration. Transcriptional profiling of the regenerating muscle suggested the matrix is dynamically remodeled [81]. Following injury, an expansion in the satellite cell pool is required. This expansion is mediated by the satellite cells themselves, who autologously remodel their local environment to induce a rapid growth in cell number and repopulate the niche through the expression of the ECM glycoprotein, Fn [82]. Fn was undetectable in the resting muscle; however, expression was detected after injury, which peaked after 5 days and proceeded to decline to baseline thereafter. Fn is a high-affinity ligand for syndecan-4 (Sdc4), a plasma membrane proteoglycan highly expressed in satellite cells [83]. Using a combination of coimmunoprecipitation (CoIP) experiments and in situ proximal ligation assays, Sdc4 was found to be a co-receptor with the Wnt receptor, Frizzled-7 (Fzd7), which formed Sdc4-Fzd7 complexes in activated satellite cells. Through a series of further CoIP experiments, the authors demonstrated that the Fzd7-Sdc4 co-receptor bound both Fn and the Wnt ligand, Wnt7a, which has previously been demonstrated to activate planar cell polarity (PCP) signaling and symmetric satellite cell divisions via the PCP effector, Vangl2 [84]. Fn was necessary to potentiate Wnt7a signaling, and this Fn-Sdc4-Fzd7-Wnt7a pathway was shown to dramatically increase the number of satellite cells present in the regenerating muscle fiber.

## Biophysical ECM Interactions

The mechanical environment of the cell has long been recognized as a key driver of growth, development, and in some cases, disease progression [85–88]. More recently, seminal work demonstrated that the elasticity of the substrate determined the fate of postnatal stem cells with soft matrices being neurogenic; stiffer matrices are myogenic and rigid matrices prove osteogenic [89]. In other studies, plating bone marrow stromal stem/progenitors on substrates that mimicked the physical properties of bone marrow arrested cell cycle progression resulting in quiescent stem cells that were reactivated when introduced to a stiff substrate [90]. Many of these studies were conducted on compliant gels, often polyacrylamides, which are then coated with an ECM protein. Control over bulk stiffness is achieved by altering the cross-linking density. However, stiffness is not a completely independent variable as the cross-linking density also affects substrate porosity and, subsequently, the anchoring of ECM proteins [91]. Mechanistically, the actin cytoskeleton is implicated, and in particular, the small GTPase RhoA/ROCK signaling pathway which has established itself as a critical node in mechanosensing. On stiff materials, tension-induced RhoA activates the nuclear factor YAP (yes-associated protein) and TAZ (transcriptional co-activator with PDZ-binding motif) which then translocate to the nucleus and regulate the differentiation of bone marrow stromal stem/progenitor cells (BMSCs) [92]. Knockdown of YAP and TAZ inhibited the differentiation of BMSCs to osteoblasts on stiff substrates suggesting YAP and TAZ are key mediators of ECM stiffness.

YAP and TAZ have also been suggested to be necessary for maintaining pluripotency, with YAP inactivated during ES cell differentiation [93]. Thus, stiff materials were reported to support proliferation and long-term self-renewal of pluripotent cells, mediated by the nuclear localization of YAP [94]. When cultured on compliant substrates, nuclear localization of YAP was inhibited and even in the presence of soluble pluripotency factors, ES cells differentiated to Tuj1+ neurons [95]. These findings could be recapitulated on stiff substrates by inhibiting F-actin polymerization or by lentiviral-mediated RNAi knockdown of YAP. Compliant substrates used for the production of motor neurons resulted in a far greater purity and yield when compared with conventional tissue-culture plastic or stiff substrates [96].

Whilst matrix stiffness affects stem cells in culture, manipulating the local mechanical environment in vivo is challenging, and thus, there is little in vivo evidence to suggest the mechanical properties of the embryonic or adult niche support stem cell maintenance or differentiation. Further, there is little quantitative, descriptive data of the local mechanical environment surrounding stem cells in a quiescent or reactive niche. Therefore, interpreting the precise nature and role of matrix stiffness on stem cells is difficult. Nevertheless, a degree of in vivo relevance was provided using MuSCs. Freshly isolated MuSC are notoriously difficult to expand in vitro [97]. The authors isolated primary MuSCs and plated them on a tuneable polyethylene glycol (PEG) hydrogel platform that consisted of multiple rigidities, including that of the skeletal muscle [98]. Using single-cell tracking software, the fate of individual cells was monitored. MuSCs plated on the soft matrices were able to expand much more rapidly than on the hard matrices. This was primarily an effect of cell survival as the stiff substrates induced significant cell death. The expanded cells were then assessed functionally in vivo. The cells expanded on soft matrices were able to engraft efficiently, particularly cells from the PEG formulation that matched the elasticity of skeletal muscle. MuSCs cultured on stiff substrates lost their potency and failed to engraft. The authors further expanded on this study by demonstrating that the biophysical environment was critical in reversing the reduced function of MuSCs from aged mice [99••]. It is well established that the regenerative capacity is reduced in MuSCs from aged mice [100]. This defect was shown to be intrinsic and not due to the aged microenvironment and could be overcome by a combination of p38/MAPK inhibition and culturing on soft hydrogel with mechanical properties matching that of juvenile muscle tissue [99••]. Importantly, it was the synergistic effects of both the biochemical and biophysical treatments that were able to generate a population of stem cells from aged mice that was able to restore strength to injured muscles in aged mice. These two studies elegantly provided in vivo functional relevance of substrate stiffness.

Despite the lack of in vivo evidence, technological developments in engineered microenvironments have helped to elucidate some interesting matrix biology discoveries. Single-cell, patterned, microenvironments were used to examine the effect of cell spreading on epidermal stem cells. On small, 20-μm diameter islands, cells differentiated into keratinocytes and remained rounded at higher frequencies than on large diameter islands (50 μm) [101]. Neither the concentration nor composition of the ECM was significant, with the effect being attributed to serum-response factor (SRF) transcriptional activity. The degree of spreading of the cells altered the G- and F-actin ratio with a reduction in the ratio releasing MAL (megakaryocytic acute leukemia protein), which is then able to relocalize and act as a cofactor for SRF. These two proteins then induce the expression of target genes FOS and JUNB that are required for differentiation. BMSCs were also found to be responsive to cell shape; however, whereas epidermal stem cells had a simple decision of either differentiating or not, the shape of BMSCs determines the lineage that they will commit to. BMSCs allowed to flatten and spread favored osteogenesis, whereas round cells underwent adipogenesis [102]. More complex shapes with varying aspect ratios have also been investigated [103]. The effects appear to be mediated by RhoA/ROCK signaling with geometries that induce actomyosin contractility promoting osteogenesis.

In addition to the classic receptor-ligand interactions by the ECM, features such as topography and geometry of the matrix substrate have been shown to modulate cell adhesion receptors and elicit a range of behaviors [104]. Using electron beam lithography, surfaces with a range of “pits” were formed. Curiously, when the pits were arranged in a disordered manner, BMSCs underwent osteogenic differentiation in vitro [105]. The level of bone mineral produced was comparable to cells cultured with osteogenic supplements. A slight adjustment in the arrangement of the pits—into a more ordered, square lattice—resulted in a substrate that was able to support BMSC growth without any spontaneous differentiation and with prolonged retention of BMSC markers and multipotency [106]. Whilst a detailed mechanism is not forthcoming, the arrangement and clustering of integrin receptors seems a likely candidate to mediate this effect [104]. It is interesting to note that the same authors also reported that a disordered surface topography induced ES cells towards a mesodermal lineage as osteogenic progenitors suggesting a developmentally conserved role for substrate architecture [107].

A chemically defined, high-throughput 3D culturing platform was used to investigate the effects of the microenvironment on pluripotency and somatic cell reprogramming [108••]. The novel approach taken allowed for the systematic analysis of a range of factors including stiffness, degradability, and biochemical composition in a combinatorial manner. Physical confinement of the cells in the 3D environment was shown to boost the reprogramming efficiency through an accelerated mesenchymal-to-epithelial transition and increased epigenetic remodeling, providing evidence that biophysical cues can modulate the epigenetic state during reprogramming [108••]. This study demonstrated the power of high-throughput technologies in uncovering unexpected phenotypes.

## Conclusions

The ubiquity of its expression and the multiple modes by which it operates render the ECM as a crucial regulator of cell and tissue behavior. This review has provided an exemplar of the different modes of ECM signaling during development, tissue homoeostasis, and regeneration in a number of stem cell niches. Despite some impressive discoveries, detailed studies of ECM interactions have failed to keep pace with the giant strides made in other areas of stem cell biology. In part, this could be attributed to difficulties in using animal models, which have proved to be an invaluable resource in stem cell biology. The pleiotropic nature of matrix signaling often results in embryonic lethality. Alternatively, disrupting ECM interactions can result in a loss of adhesion leading to a loss of tissue integrity. Where animals do survive, loss-of-function studies often fail to reveal a phenotype; however, this does not necessarily imply a lack of function. Rather, the function could be masked by genetic redundancy or compensation by other ECM molecules or isoforms. Further, many ECM proteins work synergistically with others in complex networks capable of maintaining function even when one member of the network is lost. Gain-of-function or dominant negative mutations may be better placed to provide insights into matrix protein interactions.

Recent advances in bioengineering and the development of artificial niches in vitro have provided some revealing insights into the function of the ECM—particularly the physical properties of the matrix. It would be interesting to combine traditional ligand presentation with these physical factors and take a combinatorial approach to cell-matrix interactions. The development of high-throughput arrays is well placed to meet this challenge. Further advances in three-dimensional culturing platforms and dynamic materials that are able to modulate their properties over time are exciting developments and should yield interesting insights into ECM biology [109–111]. An improved understanding of ECM signaling in the stem cell niche is vital not just in understanding the finely balanced steady state that exists in many niches but also as a potential therapeutic target which could promote endogenous stem cell repair and in the burgeoning field of tissue engineering and the development of synthetic ECM scaffolds.
